# Germline variations at *JAK2*, *TERT*, *HBS1L-MYB* and *MECOM* and the risk of myeloproliferative neoplasms in Taiwanese population

**DOI:** 10.18632/oncotarget.19211

**Published:** 2017-07-12

**Authors:** Yi-Hao Chiang, Yu-Cheng Chang, Huan-Chau Lin, Ling Huang, Chun-Chia Cheng, Wei-Ting Wang, Hung-I Cheng, Nai-Wen Su, Caleb Gon-Shen Chen, Johnson Lin, Yi-Fang Chang, Ming-Chih Chang, Ruey-Kuen Hsieh, Wen-Chien Chou, Ken-Hong Lim, Yuan-Yeh Kuo

**Affiliations:** ^1^ Division of Hematology and Oncology, Department of Internal Medicine, MacKay Memorial Hospital, Taipei, Taiwan; ^2^ Laboratory of Good Clinical Research Center, Department of Medical Research, MacKay Memorial Hospital, New Taipei City, Taiwan; ^3^ Department of Medicine, MacKay Medical College, New Taipei City, Taiwan; ^4^ Division of Hematology and Oncology, Department of Internal Medicine, MacKay Memorial Hospital, Hsinchu, Taiwan; ^5^ Institute of Molecular and Cellular Biology, National Tsing-Hua University, Hsinchu, Taiwan; ^6^ Division of Hematology, Department of Internal Medicine, National Taiwan University Hospital, College of Medicine, National Taiwan University, Taipei, Taiwan; ^7^ Department of Laboratory Medicine, National Taiwan University Hospital, Taipei, Taiwan; ^8^ Graduate Institute of Oncology, National Taiwan University College of Medicine, Taipei, Taiwan

**Keywords:** JAK2, TERT, myeloproliferative neoplasms, single nucleotide polymorphism

## Abstract

Germline variations at *JAK2*, *TERT*, *HBS1L*-*MYB* and *MECOM* have been found to associate with myeloproliferative neoplasms (MPNs) in European populations. Whether these germline variations are associated with MPNs in Taiwanese population is obscure. Here we aimed to evaluate the association of five germline variations (*JAK2* 46/1 haplotype tagged by rs12343867, *JAK2* intron 8 rs12339666, *TERT* rs2736100, *HBS1L*-*MYB* rs9376092 and *MECOM* rs2201862) and the risk of MPNs in Taiwanese population. A total of 178 MPN patients (109 essential thrombocythemia, 54 polycythemia vera and 15 primary myelofibrosis) were enrolled into this study. The information of 17033 control subjects was obtained from Taiwan Biobank database. The *JAK2* 46/1 haplotype, *JAK2* rs12339666 and *TERT* rs2736100 were significantly associated with Taiwanese MPNs (*P* = 3.6×10^-19^, 1.9×10^-19^ and 3.1×10^-6^, respectively), and *JAK2*^V617F^-positive MPNs (n=121) (*P* = 5.6×10^-21^, 4.4×10^-21^ and 8.6×10^-7^, respectively). In *JAK2*^V617F^-negative cases (n=55), only the *JAK2* 46/1 haplotype and *JAK2* rs12339666 remained statistically significant (*P*= 0.009 and 0.007, respectively). When stratified by disease subtypes, the *JAK2* 46/1 haplotype and *JAK2* rs12339666 were significantly associated with all three MPN subtypes, but *TERT* rs2736100 was only associated with essential thrombocythemia and polycythemia vera. We did not find any association of these five SNPs with *CALR* mutations in our cohort. Furthermore, the risk alleles of *MECOM* rs2201862 and *HBS1L*-*MYB* rs9376092 were demonstrated to be negatively associated with the risk of developing polycythemia vera. In conclusion, germline variations at *JAK2* (both the 46/1 haplotype and rs12339666) and *TERT* rs2736100 were associated with MPNs in Taiwanese population.

## INTRODUCTION

*The classic BCR-ABL1*-negative myeloproliferative neoplasms (MPNs) are clonal hematopoietic stem cell diseases, including three major disease entities: polycythemia vera (PV), essential thrombocythemia (ET) and primary myelofibrosis (PMF) [1]. MPNs are characterized by the overproduction of myeloid cells derived from one or more lineages. Among the three subtypes of MPNs, PV and ET are characterized by excessive formation of mature red blood cells and platelets, respectively, while PMF appears as fibrosis of the bone marrow, abnormal megakaryocytic proliferation and clustering, extramedullary hematopoiesis and variable peripheral blood counts. Somatic mutations in *JAK2*^V617F^, *CALR* exon 9 and *MPL* exon 10 have been demonstrated as three major driver mutations in MPNs, are usually mutually exclusive in the majority of cases, and involve in the activation of JAK-STAT signaling [2, 3]. The frequency of *JAK2*^V617F^ mutation is over 95% in PV, and about 60% in ET and PMF. *MPL* exon 10 mutation is found in about 1% of ET and 5% of PMF patients. In ET or PMF patients without gene mutations in either *JAK2* or *MPL*, *CALR* mutations have been observed in approximately 70% of these patients [4–8].

From both family studies and epidemiological data, inherited factors have been proposed to predispose to MPNs, and it has also been suggested that inherited single-nucleotide polymorphisms (SNPs) within *JAK2* are associated with specific MPN subtypes [9–12]. A recent study has indicated that germline variations at *JAK2* are linked to the acquisition of *JAK2* ^V617F^ mutation in MPNs [10]. Several studies have discovered that a specific *JAK2* haplotype, called ‘46/1’ or ‘GGCC’ strongly predisposes to *JAK2*^V617F^-positive MPNs in Caucasian populations [9–11]. The *JAK2* 46/1 haplotype is also demonstrated to predispose to MPNs with *MPL* mutation and PV with *JAK2* exon 12 mutations [13, 14]. In addition, two *JAK2* SNPs (rs12343867 and rs12340895) are demonstrated to express in complete linkage disequilibrium with *JAK2* 46/1 haplotype and have been used to tag this haplotype. Moreover, *JAK2*^V617F^ mutation was also found to arise preferentially on the *JAK2* 46/1 haplotype [9]. Following these observations, germline variation at *TERT* rs2736100 has also been found to associate with MPNs in Icelandic population [15], and this finding was then confirmed by others [16–18].

In order to characterize the predisposition of inherited factors towards MPNs, Tapper *et al*. have performed a genome-wide association study in European populations [17]. After meta-analysis, they found that *JAK2* rs12339666 and *MECOM* rs2201862 are significantly associated with *JAK2*^V617F^-negative MPNs. When *JAK2*^V617F^-positive cases were included in the analysis, two additional SNPs, *TERT* rs2736100 and *HBS1L*-*MYB* rs9376092, also achieved statistically significance. They also showed that *HBS1L*-*MYB* rs9376092 has a stronger effect on *JAK2*^V617F^-negative cases with *CALR* and/or *MPL* mutations, and has a stronger association with ET rather than with PV in *JAK2*^V617F^-positive cases. They concluded that multiple germline variants predispose to MPNs and linked constitutional differences in *MYB* expression to disease phenotype [17]. Although the association of *JAK2* 46/1 haplotype with MPNs has been validated across different ethnic groups, whether the other four SNPs (rs12339666, rs2736100, rs9376092 and rs2201862) might also associate with MPNs in Taiwanese population is currently unclear.

High-resolution melting analysis (HRMA) is a closed-tube and polymerase chain reaction (PCR)-based technique for the detection of gene polymorphism and mutations by measuring changes in the melting of a DNA duplex [19]. HRMA has been used for the detection and prescreening of SNPs and/or mutations including *JAK2*^V617F^ and *JAK2* exon 12 mutations in MPNs [20–22]. We have utilized HRMA for rapid and sensitive detection of *CALR* exon 9 mutations in ET using the CFX Connect real-time system (Bio-Rad Laboratories, Hercules, CA, USA) [23]. Here we aimed to determine the association of the above mentioned five SNPs with MPNs in Taiwanese population using HRMA and/or Sanger sequencing.

## RESULTS

### Mutational screening

In this cohort, *JAK2*^V617F^ mutation was detected in 38 (70.4%) of 54 PV, 76 (69.7%) of 109 ET and 9 (60%) of 15 PMF, respectively. *CALR* mutations were detected in 17 (15.6%) of 109 ET including four types of *CALR* mutants: 5 type 1 (p.L367fs*46), 8 type 2 (p.K385fs*47), 3 type 3 (p.L367fs*48) and 1 other type (p.E369fs*50). Two (13.3%) of 15 PMF harbored *CALR* mutations including two types of *CALR* mutants: 1 type 34 (p.K385fs*47) and 1 other type (p.L367fs*43). Interestingly, two (1.8%) ET patients with type 1 *CALR* mutation were also found to harbor *JAK2*^V617F^ mutation. No *CALR* mutation was detected in PV. Only one heterozygous *MPL*^W515K^ mutation was detected in 1 (0.9%) of 109 ET.

### Association of the 5 SNPs with MPN patients

**Figure 1:**
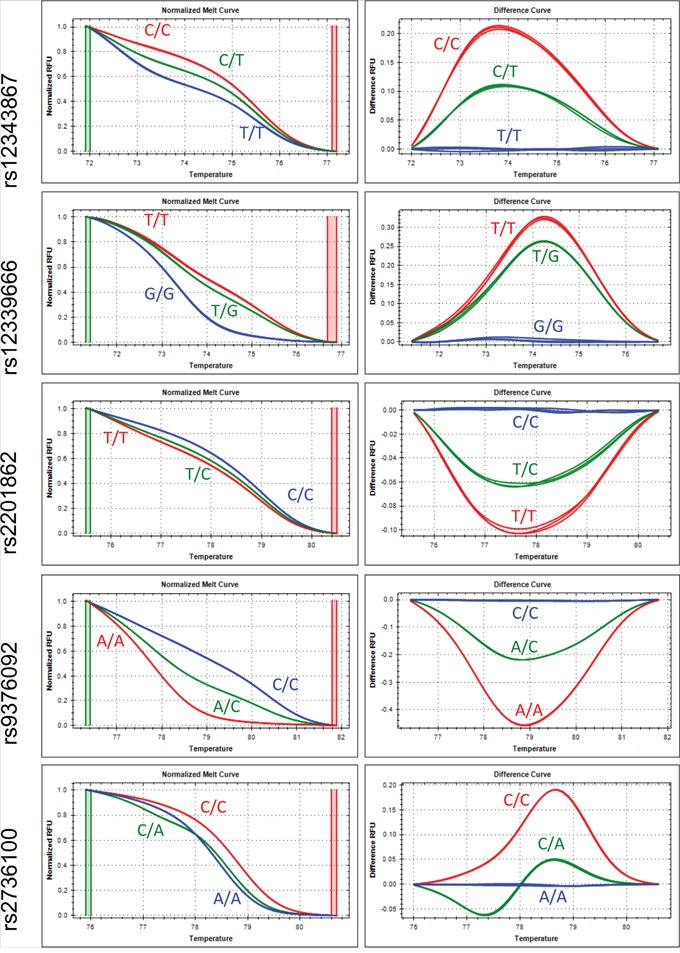
Normalized melt curves and difference curves of the 5 SNPs The curves of risk homozygosity, heterozygosity and non-risk homozygosity were shown in red, green and blue colors, respectively.

By using our HRMA platform, we have successfully genotyped the 5 SNPs including rs12343867 (*JAK2* 46/1 haplotype), *JAK2* rs12339666, *TERT* rs2736100, *MECOM* rs2201862 and *HBS1L*-*MYB* rs9376092 in more than 95% of samples from MPN patients (Figure 1). Based on the data from Taiwan Biobank, the risk allele frequency (RAF) of *MECOM* rs2201862 and *TERT* rs2736100 is significantly lower in the Taiwanese population (0.23 and 0.41, respectively) than that of the Western populations (0.48 and 0.51, respectively) indicating primordially ethnic difference in these 2 SNPs (Table 1). We found that *JAK2* rs12343867, *JAK2* rs12339666 and *TERT* rs2736100 were associated with MPNs in Taiwanese population. These 3 SNPs had stronger association with *JAK2*^V617F^-positive MPNs when compared to controls. In *JAK2*^V617F^-negative MPNs, only *JAK2* rs12343867 and *JAK2* rs12339666 remained statistically significant. Although *JAK2* rs12339666 had a modest population attributable risk (PAR) in Western MPNs (27.5%), it had the highest PAR in Taiwanese MPNs (45.8%). We did not find any association of the 5 SNPs with *CALR* mutations in our cohort possibly because of small sample sizes (n = 17).

**Table 1:** Association of the five SNPs in MPN patients stratified by mutation profiles

SNP	Alleles*	Taiwanese RAF^#^	Gene	All MPN cases (n=178^†^)			*JAK2*^V617F^-positive cases (n=121^†^)			*JAK2*^V617F^-negative cases (n=55)			*CALR*-mutated cases (n=17)			Western RAF^#^	Western *JAK2*^V617F^-negative and all *JAK2*^V617F^ positive		
				*P* value	OR (95% CI)	PAR	*P* value	OR (95% CI)	PAR	*P* value	OR (95% CI)	PAR	*P* value	OR (95% CI)	PAR		*P* value	OR (95% CI)	PAR
rs12343867	C/T	0.25	*JAK2* 46/1	3.616 × 10^-19^	2.81 (2.23 - 3.54)	42.9	5.604 × 10^-21^	3.52 (2.67 - 4.63)	46.6	0.009	1.70 (1.14 - 2.54)	36.3	0.573	1.24 (0.59 - 2.61)	28.6	0.24**^§^**	6.379 × 10^-43^	3.19 (2.69 - 3.79)	48.1
rs12339666	T/G	0.26	*JAK2* intron 8	1.941 × 10^-19^	2.81 (2.23 - 3.53)	45.8	4.365 × 10^-21^	3.50 (2.67 - 4.60)	49.1	0.007	1.71 (1.15 - 2.55)	40.2	0.701	1.16 (0.55 - 2.44)	25.1	0.26	2.287 × 10^-62^	1.76 (1.65–1.88)	27.5
rs2201862	T/C	0.23	-*MECOM*	0.316	0.87 (0.66 - 1.15)	-8.3	0.345	0.85 (0.61 - 1.19)	-10.5	0.796	0.94 (0.59 - 1.50)	-1.4	0.752	0.87 (0.38 - 2.02)	0.3	0.48	4.075 × 10^-10^	0.84 (0.80–0.89)	14.3
rs9376092	A/C	0.26	*HBS1L*-*MYB*	0.654	1.06 (0.83 - 1.34)	-0.4	0.532	1.09 (0.82 - 1.45)	0.6	0.912	0.98 (0.64 - 1.50)	-2.8	0.101	1.77 (0.89 - 3.53)	35.6	0.27	5.547 × 10^-7^	1.16 (1.09–1.23)	6.9
rs2736100	C/A	0.41	*TERT*	3.115 × 10^-6^	1.64 (1.33 - 2.02)	28.6	8.624 × 10^-7^	1.88 (1.45 - 2.42)	37.0	0.301	1.22 (0.84 - 1.78)	7.6	0.444	1.30 (0.66 - 2.55)	17.0	0.51	3.667 × 10^-26^	1.51 (1.40–1.63)	42.7

Abbreviations: CI, confident interval; MPN, myeloproliferative neoplasm; OR, odds ratio; PAR, population attributable risks; RAF, risk allele frequency; SNP, single nucleotide polymorphism.

*Risk-associated/non-risk-associated alleles.

^#^RAF for healthy controls was used to calculate the PAR.

^†^Two cases and one case of *JAK2*^V617F^-positive essential thrombocythemia were absent in rs12343867 and rs2201862 groups, respectively due to lack of genomic DNA.

^§^The information was from another *JAK2* 46/1 tagged SNP rs12340895 instead of rs12343867.

*P* values were analyzed using Pearson χ^2^-test (two-tailed) or Fisher's exact test (two-tailed).

To determine whether the 5 SNPs predispose to different subtypes of MPNs, we then analyzed the association of the 5 SNPs with subtypes of MPNs (Table 2). *JAK2* rs12343867 and *JAK2* rs12339666 were significantly positively associated with all three MPN subtypes; *TERT* rs2736100 with PV and ET; and *MECOM* rs2201862 only with MF. Unexpectedly, *MECOM* rs2201862 and *HBS1L*-*MYB* rs9376092 were found to have negative association with Taiwanese PV patients, and both had a significantly negative PAR (-27.8% and -24.9%, respectively). Among these 5 SNPs, *JAK2* rs12339666 had the highest PAR especially in PV patients (65.2%). Similar findings were found when we used the genotype distribution to calculate the association of these 5 SNPs with Taiwanese MPN patients (Supplementary Table 1).

**Table 2:** Association of the five SNPs in MPN patients stratified by disease subtypes

SNP	Alleles*	Taiwanese RAF^#^	Gene	PV (n=54)			ET (n=109^†^)			MF (n=15)			All ET and MF cases (n=124^†^)		
				*P* value	OR (95% CI)	PAR	*P* value	OR (95% CI)	PAR	*P* value	OR (95% CI)	PAR	*P* value	OR (95% CI)	PAR
rs12343867	C/T	0.25	*JAK2* 46/1	7.800 × 10^-18^	5.05 (3.38 - 7.56)	62.7	2.566 × 10^-7^	2.12 (1.58 - 2.83)	37.0	0.007	2.60 (1.26 - 5.37)	14.2	1.556 × 10^-8^	2.17 (1.65 - 2.85)	34.3
rs12339666	T/G	0.26	*JAK2* intron 8	1.345 × 10^-18^	5.33 (3.54 - 8.02)	65.2	9.319 × 10^-7^	2.03 (1.52 - 2.70)	37.5	0.001	3.17 (1.54 - 6.55)	37.6	1.892 × 10^-8^	2.14 (1.63 - 2.81)	37.5
rs2201862	T/C	0.23	-*MECOM*	0.051	0.59 (0.34 - 1.01)	-27.8	0.539	0.90 (0.64 - 1.27)	-4.2	0.075	1.95 (0.92 - 4.13)	32.6	0.980	1.00 (0.73 - 1.38)	0.3
rs9376092	A/C	0.26	*HBS1L*-*MYB*	0.049	0.61 (0.37 - 1.00)	-24.9	0.077	1.30 (0.97 - 1.73)	11.3	0.610	1.22 (0.56 - 2.68)	2.8	0.066	1.29 (0.98 - 1.69)	10.3
rs2736100	C/A	0.41	*TERT*	7.957 × 10^-4^	1.90 (1.30 - 2.78)	42.5	7.646 × 10^-4^	1.57 (1.21 - 2.06)	24.9	0.499	1.28 (0.62 - 2.62)	5.8	7.039 × 10^-4^	1.54 (1.20 - 1.97)	22.6

CI, confident interval; ET, essential thrombocythemia; MPN, myeloproliferative neoplasm; MF; myelofibrosis; PV, polycythemia vera; OR, odds ratio; PAR, population attributable risks; RAF, risk allele frequency; SNP, single nucleotide polymorphism.

*Risk-associated/non-risk-associated alleles.

^#^RAF for healthy controls was used to calculate the PAR.

^†^Two cases and one case of *JAK2*^V617F^-positive ET were absent in rs12343867 and rs2201862 groups, respectively due to lack of genomic DNA.

*P* values were analyzed using Pearson χ^2^-test (two-tailed) or Fisher's exact test (two-tailed).

### Comparison of the minor allele frequency of the 5 SNPs in MPN subtypes

We found that the minor allele frequency (MAF) of *JAK2* rs12343867 and *JAK2* rs12339666 was significantly higher in PV when all MPNs were included (Table 3) and also in *JAK2*^V617F^-positive cases (Table 4). The MAF of *MECOM* rs2201862 and *HBS1L*-*MYB* rs9376092 was significantly higher in MF only when all MPNs were included. The MAF of *TERT* rs2736100 did not have statistical difference among the three MPN subtypes. In disease subtype comparison, the MAF of *JAK2* rs12343867 and *JAK2* rs12339666 was significantly higher in PV than ET, and the MAF of *MECOM* rs2201862 was significantly higher in MF than PV when all MPNs were included (Table 3) and also in *JAK2*^V617F^-positive cases (Table 4). The MAF of *MECOM* rs2201862 and *HBS1L*-*MYB* rs9376092 in ET was significantly lower than that of MF and PV, respectively, only when all MPNs were considered.

**Table 3:** Comparison of the minor allele frequency of the 5 SNPs in MPN patients

SNP	Gene	Alleles*	PV MAF (n=54)	ET MAF (n=109^†^)	MF MAF (n=15)	PV vs. ET		PV vs. MF		ET vs. MF		PV vs. ET vs. MF
						*P* value	OR (95% CI)	*P* value	OR (95% CI)	*P* value	OR (95% CI)	*P* value
rs12343867	*JAK2* 46/1	C/T	0.63	0.42	0.47	<0.001	2.39 (1.48-3.84)	0.108	1.94 (0.86-4.40)	0.598	0.81 (0.38-1.75)	0.001
rs12339666	*JAK2* intron 8	T/G	0.66	0.42	0.53	<0.001	2.63 (1.63-4.25)	0.213	1.68 (0.74-3.81)	0.249	0.64 (0.30-1.37)	<0.001
rs2201862	-*MECOM*	T/C	0.15	0.21	0.37	0.179	0.65 (0.35-1.22)	0.008	0.30 (0.12-0.75)	0.056	0.46 (0.20-1.04)	0.030
rs9376092	*HBS1L*-*MYB*	A/C	0.18	0.31	0.30	0.009	0.47 (0.27-0.83)	0.135	0.50 (0.20-1.26)	0.895	1.06 (0.46-2.43)	0.031
rs2736100	*TERT*	C/A	0.56	0.52	0.47	0.429	1.21 (0.76-1.92)	0.340	1.48 (0.66-3.34)	0.595	1.23 (0.57-2.64)	0.571

CI, confident interval; ET, essential thrombocythemia; MAF, minor allele frequency; MPN, myeloproliferative neoplasm; MF; myelofibrosis; PV, polycythemia vera; OR, odds ratio; SNP, single nucleotide polymorphism.

*Risk-associated/non-risk-associated alleles.

^†^Two cases and one case with ET were absent in rs12343867 group and rs2201862 group, respectively due to lack of sample.

*P* values were analyzed using Pearson χ^2^-test (two-tailed).

**Table 4:** Comparison of the minor allele frequency of the 5 SNPs in *JAK2*^V617F^-positive MPN patients

SNP	Gene	Alleles*	PV MAF (n=38)	ET MAF (n=74^†^)	MF MAF (n=9)	PV vs. ET		PV vs. MF		ET vs. MF		PV vs. ET vs. MF
						*P* value	OR (95% CI)	*P* value	OR (95% CI)	*P* value	OR (95% CI)	*P* value
rs12343867	*JAK2* 46/1	C/T	0.72	0.45	0.50	<0.001	3.18 (1.75-5.80)	0.067	2.62 (0.91-7.50)	0.696	0.82 (0.31-2.19)	<0.001
rs12339666	*JAK2* intron 8	T/G	0.76	0.46	0.50	1.437 × 10^-5^	3.79 (2.04-7.05)	0.027	3.22 (1.11-9.34)	0.745	0.85 (0.32-2.26)	7.334 × 10^-5^
rs2201862	-*MECOM*	T/C	0.14	0.22	0.33	0.206	0.62 (0.29-1.31)	0.087	0.34 (0.11-1.09)	0.250	0.55 (0.19-1.58)	0.161
rs9376092	*HBS1L*-*MYB*	A/C	0.22	0.31	0.22	0.170	0.64 (0.34-1.21)	1.000	1.01 (0.29-3.47)	0.439	1.58 (0.49-5.06)	0.363
rs2736100	*TERT*	C/A	0.59	0.56	0.44	0.654	1.14 (0.65-1.99)	0.256	1.81 (0.64-5.11)	0.349	1.60 (0.60-4.27)	0.524

CI, confident interval; ET, essential thrombocythemia; MAF, minor allele frequency; MPN, myeloproliferative neoplasm; MF; myelofibrosis; PV, polycythemia vera; OR, odds ratio; SNP, single nucleotide polymorphism.

*Risk-associated/non-risk-associated alleles.

^†^Two cases and one case with ET were absent in rs12343867 group and rs2201862 group, respectively due to lack of sample.

*P* values were analyzed using Pearson χ^2^-test (two-tailed) or Fisher's exact test (two-tailed).

### Linkage of *JAK2*^V617F^ mutation and *JAK2* 46/1 haplotype

We also evaluated the location of *JAK2*^V617F^ mutation in heterozygous *JAK2* 46/1 haplotype cases (Table 5). 38 patients (9 PV and 27 ET) were found to have heterozygous *JAK2*^V617F^ mutation in our cohort. Consistent with previous studies [9–11, 24], the frequency of *JAK2*^V617F^ mutation was significantly higher on the 46/1 allele (86%, *P* = 7.428 × 10^-6^) in this cohort. Similar findings were also found when PV and ET patients were evaluated separately.

**Table 5:** Location of *JAK2*^V617F^ mutation in heterozygous *JAK2* 46/1 haplotype MPN patients

Category	Case no.	On 46/1 allele	*P* value
All MPNs	37	32 (86%)	7.428 × 10^-6^
PV	9	8 (89%)	0.039
ET	27	23 (85%)	3.107 × 10^-4^

ET, essential thrombocythemia; MPN, myeloproliferative neoplasm; no., number; PV, polycythemia vera.

### Clinical and prognostic implications of the five SNPs and the use of cytoreductive therapy

In MPN patients harbored the risk alleles of rs12343867C (*JAK2* 46/1) and rs12339666T (*JAK2* intron 8), more patients were found to have splenomegaly at diagnosis (C vs T: 60% vs 40%, *P* = 0.007; T vs G: 61% vs 39%, *P* = 0.011, respectively), and fewer patients had overall hemorrhagic complications (C vs T: 36% vs 64%, *P* = 0.007; T vs G: 38.6% vs 61.4%, *P* = 0.012, respectively) (Supplementary Table 2). MPN patients harbored the risk allele of *MECOM* rs2201862T were found to have significantly less myelofibrosis transformation (T vs C: 42.9% vs 57.1%, *P* = 0.032). In MPN patients carried the risk allele of *HBS1L*-*MYB* rs9376092A, fewer patients had overall thrombotic complications (A vs C: 37% vs 63%, *P* = 0.012). Besides, those MPN patients with homozygous rs9376092A risk allele had encountered significantly fewer overall hemorrhagic complications (AA vs AC vs CC: 6.8% vs 52.3% vs 40.9%, *P* = 0.043). In patients harbored the *TERT* rs2736100C risk allele, significantly more patients were diagnosed at age less than 60-year-old (C vs A: 59.2% vs 40.8%, *P* = 0.008). Regarding to the use of cytoreductive therapy in MPN patients, significantly more patients with age equal to or higher than 60-year-old were treated with cytoreductive therapy (yes vs no: 87.5% vs 12.5%, *P* = 0.043).

## DISCUSSION

In our study, we evaluated five MPN-associated SNPs in Taiwanese MPN patients, and identified significant associations of 3 of the 5 SNPs, rs12343867 (*JAK2* 46/1), rs12339666 (*JAK2* intron 8) and rs2736100 (*TERT*) in our cohorts. Our results showed that the associations of these 3 germline variations with MPNs in our cohort are similar to those observed in Western populations including stronger associations for rs12343867 and rs12339666 in PV patients. Consistent with the Western populations, the *JAK2* 46/1 haplotype rs12343867 and *JAK2* intron 8 rs12339666 had the greatest effect on *JAK2*^V617F^-positive and *JAK2*^V617F^-negative MPN in Taiwanese population. Our data also confirmed the associations of germline variations at *JAK2* and *TERT* with MPNs in Taiwanese population.

Furthermore, both *MECOM* rs2201862 and *HBS1L*-*MYB* rs9376092 were not found to be a risk factor for Taiwanese MPNs in our study, although they have been demonstrated to have moderate association with Western MPNs. This discrepancy in *MECOM* rs2201862 might be explained by the difference in the RAF for healthy controls between the Western (0.48) and Taiwanese populations (0.23) (Table 1). For *HBS1L*-*MYB* rs9376092, we might not be able to detect the modest association of this SNP with Taiwanese MPNs due to a relatively small patient size in our study. However, both *MECOM* rs2201862 and *HBS1L*-*MYB* rs9376092 were found to have negative association with PV in our cohort. These findings argue that different genetic background might account for the discrepancy seen between Western and Taiwanese populations in these 2 SNPs rather than sample size. Our observations suggested that *MECOM* rs2201862 and *HBS1L*-*MYB* rs9376092 might have different contributions to the development of MPNs in different ethnic groups.

Regarding to the clinical and prognostic implications of the 5 SNPs in MPN patients, our results showed that MPN patients harbored the risk alleles of rs12343867_C (*JAK2* 46/1) and rs12339666_T (*JAK2* intron 8) were more likely to have splenomegaly at diagnosis and were less likely to encounter hemorrhagic complications. Besides, those MPN patients with homozygous rs9376092_A (*HBS1L*-*MYB*) risk allele were also found to have fewer hemorrhagic complications. Furthermore, MPN patients carried the risk allele of rs9376092_A were also found to have fewer thrombotic complications. Only the risk allele of *MECOM* rs2201862_T was found to have protective effect on myelofibrosis transformation. To the best of our knowledge, these clinical and prognostic implications of the above-mentioned 4 SNPs have never been reported in MPNs. Whether the harboring of risk allele(s) of these 4 SNPs might have any therapeutic implications requires further study. Moreover, the cause(s) of the protective effects of these 4 risk alleles against hemorrhagic and/or thrombotic complications, or myelofibrosis transformation are not yet clear, and further study is also warranted to confirm our observation. In contrast to the findings of Krahling *et al*. from Hungarian population that *TERT* rs2736100_C risk allele predisposes to the development of MPNs with the co-occurrence of solid tumors, especially with the usage of cytoreductive treatment [25], we found that MPN patients harboring the *TERT* rs2736100_C risk allele were only associated with younger age at diagnosis. In addition, the use of cytoreductive therapy was not associated with the risk of secondary solid cancer in our cohort, but was more frequently seen in older patients indicating the compliance with the current treatment recommendation. More studies from different populations may be needed to clarify the discordance between our results from that of Krahling *et al*.

In addition to the 5 SNPs screened in our study, Hinds *et al.* has recently identified additional germline variants at *TERT*, *SH2B3*, *TET2*, *ATM*, *CHEK2*, *PINT* and *GFI1B* that predispose to *JAK2*^V617F^ clonal hematopoiesis and MPNs in another Western population [26]. In their study, two *TERT* loci, rs7705526 and rs2853677, were found to be the second most statistically significant SNPs following the *JAK2* 46/1 haplotype. They also indicated that *TERT* rs2736100 is associated with MPNs, which is consistent with our findings in this study. The biological function of *TERT* rs2736100 has been reported to relate to myeloproliferative phenotypes, including increased red blood cell, platelet counts and white blood cells of the myeloid lineage [15]. Moreover, another *TET2* rs3733609 C/T genotype was recently found to associate with *JAK2*^V617F^-positive MPNs in a Chinese cohort and related to a proliferative potential on erythroid lineage [27]. The RAF of *TET2* rs3733609 in healthy subjects in the Taiwanese population (6.68%, data from the Taiwan Biobank) is similar to that in the Chinese population (6.35%, data from [27]), but different from that in the European populations (1%, data from the 1000 Genomes Project). Furthermore, it is also noteworthy that other SNPs at *JAK2* such as rs4495487 in the Japanese population and rs12342421 in the Hong Kong Chinese population have also been found to associate with MPNs [28, 29], indicating that SNPs at *JAK2* are important in MPNs as high risk factors. Because we did not evaluate *JAK2* rs4495487, *JAK2* rs12342421 and *TET2* rs3733609 in this study, whether these germline variants may also predispose to MPNs in Taiwanese population require further study.

In conclusion, we identified genetic predisposition of germline variations at *JAK2* 46/1, *JAK2* intron 8 and *TERT* to MPNs in the Taiwanese population. We also revealed negative association of germline variants at *MECOM* and *HBS1L*-*MYB* with PV in the Taiwanese population. Our study is limited by the evaluation of only 5 SNPs that have been known to associate with MPNs in Western populations. Additional germline variations that predispose to MPNs may be further discovered by genome-wide association study in Taiwanese population. Finally, the contribution of these germline variations to clonal hematopoiesis in the patients with myeloproliferative phenotypes will be worthwhile to investigate in the future.

## MATERIALS AND METHODS

### Patients

A total of 178 Taiwanese MPN patients (109 ET, 54 PV and 15 PMF) seen at MacKay Memorial Hospital from Oct 2009 to Oct 2015 were enrolled into this study. The diagnosis of MPNs was based on the 2008 World Health Organization classification. Genetic testing in MPN patients has been approved by the Institutional Review Board of MacKay Memorial Hospital. Written informed consent was obtained from patients or was waived for deceased patients when deidentified leftover samples were used as approved by the Institutional Review Board.

### SNP information in Taiwanese and Western populations

The information of 5 SNPs (rs12343867, rs12339666, rs2736100, rs9376092 and rs2201862) in Han Chinese population in Taiwan were obtained from publicly available Taiwan Biobank database (https://taiwanview.twbiobank.org.tw, last accessed on 23th August 2016). The SNPs in Taiwan Biobank database were screened by whole genome sequencing using Ion Proton (497 healthy subjects) and Illumina Hiseq (500 healthy subjects) next-generation sequencing platforms, and genome-wide genotyping using the Affymetrix Axiom-Taiwan Biobank Array Plate (16036 healthy subjects), which is a customized hybridization-based oligonucleotide array with approximately 653,291 SNPs. The number of 46/1 alleles (349 in cases and 720 in the Wellcome Trust Case Control Consortium controls) and the number of non-46/1 alleles (346 in cases and 2280 in the Wellcome Trust Case Control Consortium controls) for another *JAK2* 46/1 tagged SNP rs12340895 instead of rs12343867 in Western population were adopted from Jones *et al.* [9], and were used to calculate and compared to rs12343867 since the implication of 46/1 haplotype is identical in all *JAK2* 46/1 tagged SNPs. The information of the other SNPs in Western population was adopted from Tapper *et al.* [17] (Table 1).

### Mutational screening and SNP genotyping

All patients were screened for *JAK2*^V617F^, *CALR* and *MPL* mutations by Sanger sequencing and/or allele-specific PCR as previously described [23, 30]. Genomic DNA derived from peripheral blood, bone marrow, and/or buccal swab were extracted using the high pure PCR template preparation kit (Roche diagnostic GmbH, Mannheim, Germany) according to the manufacturer's instructions. The concentration of gDNA ranged between 16 and 20 ng/μL (in ddH_2_O) for peripheral blood and bone marrow, and between 6 and 20 ng/μL (in ddH_2_O) for buccal swab. The purity of gDNA was an A260/A280 ratio ranging between 1.7 and 2.2, and an A260/A230 ratio larger than 1.5 when measured using NanoDrop ND-1000 spectrophotometer (Thermo Fisher Scientific, Wilmington, DE). PCR amplicon of positive controls was run in a 2% agarose gel to check for the quality. The appropriated annealing temperatures for HRMA were optimized using a temperature gradient. Any aberrant amplification plots of gDNA samples detected by HRMA were re-analyzed using Sanger sequencing to determine the genotypes of the samples.

HRMA and/or Sanger sequencing were used for genotyping the 5 SNPs: rs12343867, rs12339666, rs2201862, rs9376092, and rs2736100. For HRMA, genomic DNA was amplified using the Precision Melt Supermix (Bio-Rad, Hercules, CA, USA) in the CFX Connect^TM^ Real-Time System (Bio-Rad) according to the manufacturer's instructions [13]. General primers were designed using Primer3 (http://bioinfo.ut.ee/primer3-0.4.0/), while melting temperature-shift primers were designed as previously described by other [31]. Primer sequences and annealing temperature for each primer are shown in Supplementary Table 3. For each HRMA experiment, positive genotypes that have been confirmed by Sanger sequencing were also included that the genotype of DNA samples found to have distinct melting curves from wild type could be compared and annotated.

For Sanger sequencing, a target SNP of individual genomic DNA was amplified using the GoTaq® Green Master Mix (Promega, Madison, WI, USA) followed by a pre-sequencing step using the USB ExoSAP-IT and a sequencing step using the BigDye® v3.1 (Applied Biosystems™, Carlsbad, CA, USA) in the ABI Veriti 96 Well Thermal Cycler (Applied Biosystems™) according to the manufacturer's instructions. Sequences were aligned using the CodonCode Aligner (Centerville, MA, USA).

### Linkage of *JAK2*^V617F^ and *JAK2* 46/1 haplotyope

Linkage of *JAK2*^V617F^ and *JAK2* 46/1 tagged SNP rs12343867 was detected using allele-specific PCR as previously described [9]. All novel single-nucleotide variant that was only detected once was treated as artifact and was excluded.

### Clinical and prognostic implications of the five SNPs and the use of cytoreductive therapy

The association of the 5 SNPs and the use of cytoreductive therapy with several clinical and prognostic parameters including age at diagnosis, sex, presence of splenomegaly at diagnosis, survival status, presence of secondary solid cancer before or after MPN diagnosis, presence of myelofibrosis or acute leukemia transformation, and overall hemorrhagic or thrombotic complication were evaluated. Clinical information was captured by retrospective chart review.

### Statistical analysis

We used Pearson Chi-square (two-tailed) or Fisher's exact test (two-tailed) to analyze difference between patients and healthy controls. Chi-square was used to calculate odds ratios (ORs) and 95% confidence intervals. Exact binomial distribution (two-tailed) was used to calculate deviations from expected allelic ratios of 50%. PAR was calculated as 1 − (1/[p^2^ OR_homo_ + 2p {1 − p} OR_hetero_+ {1 − p}^2^]) for all SNPs in all populations, and 1 − (1/[p^2^ OR_allele_ + 2p {1 − p} OR_allele_+ {1 − p}^2^]) was used for *JAK2* 46/1 tagged SNP in Western population. “p” is the RAF in the controls. OR_homo_ and OR_hetero_ are OR associated with risk homozygotes and heterozygotes, respectively, relative to non-risk homozygotes, and OR_allele_ is OR associated with risk allele relative to non-risk allele for *JAK2* 46/1 tagged SNP in Western population [17, 32]. Statistical analyses were performed using the Statistical Package of Social Sciences software (version 22.0; IBM, New York, NY, USA). A two-side *P* value of less than 0.05 was defined as significant difference.

## SUPPLEMENTARY MATERIALS TABLES




