# Supplementary material for: Germline variations at JAK2, TERT, HBS1L-MYB and MECOM and the risk of myeloproliferative neoplasms in Taiwanese population
Source: Oncotarget. 2017 Jul 12;8(44):76204–13. doi: 10.18632/oncotarget.19211 (PMC5652698; doi:10.18632/oncotarget.19211)
Supplement: Supplementary file 1 [file oncotarget-08-76204-s001.pdf]

Germline variations at *JAK2*, *TERT*, *HBS1L-MYB* and *MECOM* and the risk of myeloproliferative neoplasms in Taiwanese population

SUPPLEMENTARY MATERIALS

Supplementary Table 1: The distribution and the association of the five SNPs in MPN patients stratified by mutation profiles and disease subtypes

See Supplementary File 1

Supplementary Table 2: Clinical and prognostic implications of the five SNPs in 178 Taiwanese MPN patients

See Supplementary File 1

Supplementary Table 3: Primer sequences for the five SNPs used in Sanger sequencing and HRMA

SNPs	Experiment	Direction	Primer sequence (5' to 3')	Annealing temperature
rs12343867	Sanger sequencing	Forward	CTGGAATTATGAAGACAAAGCA	55-60 °C
		Reverse	GCATGGGGTACGATTTATACTT	
	HRMA	Forward	<u>**GCGGGCAGGGCGGC**</u>TTTAATGGAACTGACAGAAATGATTAC	63°C
		Forward	<u>**GCGGGC**</u>TTTAATGGAACTGACAGAAATGATTAT	
		Reverse	CTGTGAACACCTAAATTTAGCCAAAAAATA	
rs12339666	Sanger sequencing	Forward	tgggccaagaaatatgctgt	56-60 °C
		Reverse	aaaaacagggcaaacatgaga	
	HRMA	Forward	<u>**GCGGGCAGGGCGGC**</u>TGAAATTTTGTATTGAGAAGTCCAA	56 °C
		Forward	<u>**GCGGGC**</u>TGAAATTTTGTATTGAGAAGTCCAC	
		Reverse	CCTTTGTATCTAGCTTTATTTTCATTTTCT	
rs2201862	Sanger sequencing	Forward	ggaacttgctggaaactgga	60 °C
		Reverse	acatggaagctgccaagact	
	HRMA	Forward	<u>**GCGGGCAGGGCGGC**</u>AAATTTGCATAAGTAACTTGGAGCTG	63 °C
		Forward	<u>**GCGGGC**</u>AAATTTGCATAAGTAACTTGGAGCTA	
		Reverse	CCTGCAGACATTTTCCCCATTGTC	
rs9376092	Sanger sequencing	Forward	GATCACCCATCCATTCATCC	58-60 °C
		Reverse	ACCCTGCACCAGTTACATGC	
	HRMA	Forward	<u>**GCGGGCAGGGCGGC**</u>GGCCAACATTGTTCGTCTTTG	56 °C
		Forward	<u>**GCGGGC**</u>GGCCAACATTGTTCGTCTTTT	
		Reverse	TGGGAGGTAGCTCATATGAAGAAGACAGGC	
rs2736100	Sanger sequencing	Forward	GCTGTTTTCCCTGCTGACTT	56-60 °C
		Reverse	ACGTTGCTGTCACTCACTGG	
	HRMA	Forward	TGACACCCCCACAAGCTAAG	56-63 °C
		Reverse	GGGAACAAAGGAGGAAAAGC	
